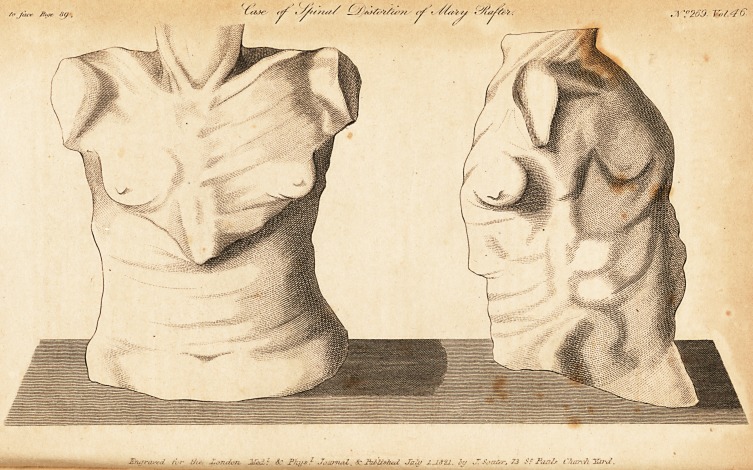# Additional Cases and Remarks, Illustrative of Spinal Diseases

**Published:** 1821-07

**Authors:** Edward Harrison

**Affiliations:** formerly President of the Royal Medical and Royal Physical Societies of Edinburgh; Corresponding Member of the Medical Society of London, &c.


### Medical and Physical Journal.

### Original Communications, Select Observations, etc.

### *Additional Cases and Remarks, illustrative of Spinal Diseases.* By EDWARD HARRISON, M.D. F.R.A.S.ED.; formerly President of the Royal Medical and Royal Physical Societies of Edinburgh; Corresponding Member of the Medical Society of London, &c.

MARY RAFTER* has enjoyed uniform good health under the present plan of treatment. Her face, which was at first thin, pale, and haggard, has become plump, blooming, and juvenile. Her complexion is good, and cheeks ruddy. She has gained a great deal of flesh. Her limbs are well-formed, and the lower ones are alike in length and appearance. The left leg was at first much shorter than the right. She walks erect, and requires no support. Her appetite is good, and her bowels have regularly performed their functions, without any artificial interference. She sleeps well, and is always cheerful. Her breathing is easy in all positions, and the fullness of the abdomen has entirely disappeared. The lumbar vertebræ, which at first were thrust down upon the sacrum, have risen higher in the back, and the arch is much reduced and has no longer any lateral turn. The five bones of which this arch consists are all loose and moveable. The transverse processes are nearly of the same level and depth below the skin on both sides. Her weight† is fifty-seven pounds.

The distortion has been gradually and regularly diminishing ever since she entered upon the present course, so as to hold out the reasonable prospect of ultimate restoration to a good

* Report continued from vol. xliv. p. 442.

† In the former part of the case, the weight, which was by mistake noted at fifty-four pounds, should have been stated at only forty-four pounds.

figure, could she have been induced to persevere long enough in the treatment. Feeling herself quite well, and being impatient of longer confinement, she was this day (May 12th,) released from all restraint at her own desire, and suffered to go home, with an assurance that, unless she submitted to remain longer in the recumbent posture, for the affected parts to recover their lost tone and healthy energy, the deformity, instead of continuing stationary, would certainly go on increasing, till she either sunk under the effects of it or was rendered unfit for any useful employment.

It is clear, from the progressive improvement which has taken place in the disposition of Mary Rafter's spine and chest,* that no bony union or true anchylosis had taken place in either of them. When that connexion is once formed, the parts can never be again disunited by any known process. The distortion, such as it is, must remain fixed and unalterable. Mary's case shows that we have in this country been led to entertain a narrow and erroneous opinion of spinal distortions and curvatures, by supposing that, when once established, the malady is always rendered permanent and incurable.

I omitted to mention in its proper place, that Mary Rafter's eyesight was very defective when she first came under my care, and had been progressively getting worse for several years. She could not see distant objects; her perception of near ones was dull, imperfect, and obscure. The sight is much improved: it has been regularly becoming clearer and brighter from the commencement of her treatment. In the third and fourth cases, the vision is reported to have grown better as the spinal disorder abated. I am preparing to communicate other cases to the public, through the medium of this Journal, in which equal benefit has been derived from similar means.

The intimate connexion which has been observed to subsist between vision and spinal maladies, may, it is conceived, be satisfactorily explained on anatomical principles. The sixth pair of nerves, the pathetic, arise, it is well known, from the corpora pyramidalia, and are expended upon the abductores oculorum. According to some pathologists, the great sympathetic derives its origin from this nerve. According to others, this nerve ascends from the great ganglion of the intercostal to join the sixth. However that may be, the two are certainly united together in the canalis caroticus. Here the sympathetic approaches the carotid artery, and accompanies it through the base of the skull and along the neck. The nerve afterwards traverses the thorax and abdomen to the lower part of the

---

* See the engraved prints. These and the former plates were taken from models cast by Mr. Mazzoni, of No. 337, Strand, and are now in my possession.

pelvis. In this long and extensive range, it communicates with, and distributes influence to, nearly every part of the human frame which is situated below the head. It is the connecting bond of the ganglionic system, and is intimately mixed with the spinal nerves.—After this short exposition, we can easily perceive a real foundation for that sympathy which is known to subsist between the eyes and several internal organs. We meet with too many examples of the appearance of symptoms remote from their source, to doubt the truth of the connexion on that ground alone. Pregnant women encounter the most distressing sensations in the feet, as if the skin were pricked with something sharp or pierced by the teeth, when the uterine nerves are irritated by the weight and distention of the gravid womb. Tumors seated within the pelvis produce great misery in both sexes, and swellings of the axilla affect the fingers in like manner. But, of all proofs, the most striking and unequivocal is drawn from the well-established fact, that distressing pains, referred to the very bottom of the foot, are often felt for a long time after the limb has been amputated above the knee of the same side. These occurrences lead us to deny the adage—*Ubi dolor ibi mali sedes.* Having shown that affections proceed from the nervous trunks to the extremities, we shall have no difficulty in maintaining that they are transmitted in a contrary direction from the minute branches, through the trunks to the brain or spinal marrow. In this way we become affected with lethargy, apoplexy, palsies, convulsions, epilepsy, and, according to some, with tetanus and hydrophobia. Since a distempered state of the nerves displays itself under so many forms, can we hesitate for a moment to believe that vertebral dislocations produce the internal pains and uneasiness with which paraplegia is constantly attended. In many other diseases, the indications and effects are likewise observed in apparently dissimilar portions of the frame. These seemingly-unconnected phenomena are held together by an impalpable principle, denominated sympathy, which regulates many of the most important operations of living animals.

In proportion as we get acquainted with this occult power and its regulating laws, we shall be better able to detect the early approaches of some of our most formidable distempers. Among these we must include paraplegia, a disease which has not hitherto received its full share of professional consideration. This malady is occasioned by some affection of the spinal marrow, which unfits its nerves from propagating their energies with due vigour to the ramifying extremities.

The seat of paraplegia is always fixed in the spine, yet the symptoms, as we have already observed, reveal themselves in distant parts of the frame. Distortions of the cervical vertebræ

occasion the most inveterate head-ach, which is equally felt within and on the outside of the skull. This arises partly from a disturbed condition of the cervical nerves in their passage to the outside of the head,—partly from obstacles interposed between the returning blood and the head, at the confluence of the internal and external jugular veins. A woman, whose spine was very crooked, *(dont l'epine etoit tres-courbee,*)* constantly suffered, after eating, a most violent pain in her left great toe, which generally went off with copious alvine discharges. Clysters increased its intensity, so long as they remained in the bowels. Many remedies were given, to no good purpose. After death, the lower false ribs on the left side were found thrust into the bottom of the belly and iliac region, and, by compressing the sigmoid flexure of the colon, prevented the free passage of excrementitious matters. The lumbar congeries of nerves squeezed between the displaced bones and feces, became disturbed in their motions. Hence arose the affection of the crural nerve, which was transmitted by the saphenal branch to the very extremity of the foot.

The anatomical communication formed in the brain between the abductores oculorum and great sympathetic, enables us to understand why pressure and wounds of this nerve, in the neck, breast, and belly, occasion convulsions in the eyes and loss of sight. We can also perceive how the irritation of intestinal worms produces contracted pupils and prominent eye-balls. For the same reason, the nerves of the eyes exercise great influence over the functions of the stomach.† It is well known that rapid circular movements of bodies often provoke vomiting; and that, in sailing swiftly along narrow rivers, sickness is excited by keeping the eyes steadily fixed upon land. The over-straining of the eyes in reading is likewise known to induce nausea in delicate persons, and sometimes vomiting. Were more proofs wanted to establish the reciprocal connexion between these nerves, they may be drawn from the well-known fact, that, after dividing the great sympathetic, the eye of the same side loses its lustre, sinks in the socket, and the pupil is dilated.

Mrs. A. B. the subject of the second case,‡ has, for some time, remained in good health. She has participated, like other ladies, in the amusements and recreations of her age and station in society.

---

* PORTAL, *Anatomie Medicale*, vol. iv. p. 277.

† The present Essay will be quickly followed by others, to show the true state of the vertebral column in spinal distortions, and their injurious effects on the spinal nerves.

‡ See *Medical and Physical Journal*, vol. xliv. p. 444.

*Third Case.*

Mr. C. D. continues to enjoy good health. The tenderness over the liver, and all the other symptoms of his former indisposition, having entirely left him, he walks about daily and alone in his father's park and grounds. This recreation is performed with ease, produces no fatigue, nor is it attended with any inconvenience.

*Fourth Case.*

The only remaining distortion under which Miss E. F. now suffers, consists in a very slight prominence and a very small obliquity of the three lower cervical, and four upper dorsal, vertebræ. She is in all other parts restored to her natural figure, which is peculiarly fine. These bones keep gradually descending, so as to afford the pleasing expectation of ultimate success at no distant period. All the ribs and the sternum have been sometime replaced. She is gone down to the sea-side to enjoy the summer months, and travelled with ease the first day more than fifty miles.

Many of the most respectable metropolitan, and several country, practitioners, among whom was Mr. Baynton, had at different times been consulted for this lady before I was called into attendance. They unanimously agreed that the displacement of the spine and ribs was irreparable.

Discouraged by this coincidence of opinion, she had ceased to apply for assistance, till chance led her to consult me. Though the exact date of her complaint cannot be clearly fixed, there is good reason to believe that it begun in the higher part of the dorsal spine, when she was only five years old. From the commencement, it kept gradually increasing till she came under my care; and her health had always been extremely delicate. The pectoral symptoms disappeared entirely soon after my first attendance, and have never recurred. Her appetite is delicate and small, but she labours under no complaint. Her menses have returned several times.—*May 20th*, 1821.

*Fifth Case.*

This youthful patient has resided constantly near the sea-coast ever since my former statement of her disorder. The accounts derived, from the mother and other friends, of her health, figure, and rapid growth, are of the most favourable description. The spine and chest are reported to have retained their proper form and appearance.

### ADDITIONAL CASES.

### *Seventh Case.*

Miss I. K. æt. 27, of a fair and sanguine complexion, has great pain and inconvenience in advancing her lower limbs. She feels uneasiness in the throat when pressure is made upon the upper part of the dorsal spine. Swallowing is often painful, and requires a great effort. Any exertion, and in particular going up stairs, has for some time been difficult, and productive of great fatigue. The descent is much easier. Her arms are generally numb, and become tremulous after slight employment of them. She is subject to frequent attacks of numbness and insensibility in her fingers and toes, which are at the time swelled and red. These symptoms, after continuing for a longer or shorter time, generally go off suddenly. During their presence the affected parts admit of being cut or bruised, without exciting pain. For a long time past she has complained of fullness and heaviness in her head, which appears to open and shut. Coughing generally produces a gush of tears from both her eyes. It is followed with temporary loss of memory, and a sensation as if she should lose her understanding.

On examination, the last cervical vertebra was observed to be too prominent. Five of the upper dorsal and four lumbar vertebræ are tender and protuberant. Her back and shoulders have been gradually growing outwards for more than a year, and now make an unsightly curve. About three years since, she hurt her back, and from that time she has always felt uneasiness and inconvenience in it after much exercise or great exertion. These symptoms have considerably increased in consequence of a fit of illness which she had in the spring. Appetite good; menses regular; bowels and pulse natural. She is five feet one and half inches in height. She underwent the usual process for half an hour this afternoon, and all the vertebræ were observed to recede from the frictions, especially that in the neck.—*Oct.* 26, 1820.

The cervical bone disappeared the first day, and she has no longer any inconvenience in swallowing. The four lumbar vertebræ have already retired into their natural situations, leaving a groove where the loins were too full. For several days afterwards, she had not the smallest ability to elevate or move her feet from her. The power is fully returned, and she now uses them with perfect freedom. All the dorsal vertebræ are much fallen. On Saturday last, the two upper dorsal bones, in particular, were observed to sink more than usual during the friction. For the remainder of that day she was troubled with violent palpitations, with great weakness of her arms and extreme debility. They are already gone, and she feels unusually well.—*Nov.* 5.

The vertebræ being still more depressed, the debility and palpitations returned, in a smaller degree, during the afternoon of Monday, after the friction of that morning; and still less in degree on Wednesday.—*Nov.* 15.

The two upper dorsal bones were this morning nearly forced into their natural places. She was, in consequence, seized with great difficulty in breathing, a strong apprehension of strangulation, and became violently hysterical. These distressing symptoms went off gradually after a few minutes of quietness. —*Dec.* 29.

All the vertebræ are entirely replaced; and it now appears that every one of them has been more or less protuberant, so that at first the spine appeared like an elevated ridge. It is now sunk into a marked groove. The unsightly fullness between the shoulders is converted into a graceful hollowness, and the small of her back is beautifully shaped. The sternum and ribs are quite restored. The health is very good. She has gained a great deal of colour, and looks extremely well. She has increased three inches, in consequence of her back getting straight, and is much heavier since she begun her cure.—*Jan.* 20, 1821.

Miss I. K. continues to enjoy the best state of health. Her figure preserves its beautiful form and shape, as when she first arose from her couch. She walks about, and erect, for several hours daily. The remainder are still passed in lying down, from a fear that the spine and chest may not yet have fully recovered their healthy actions and tone.—*May* 24, 1821.

*Eighth Case.*

Master James Walker, aged twelve years last December, of a sanguine temperament, fair complexion, and stout make, is completely paraplegic in his right leg and thigh. They are both generally cold to the touch. The skin looks very motley and reddish from the hip downwards. Sensibility is much impaired. The limb is somewhat extenuated, and hangs lower than the other. The flesh is nearly as firm and hard as on the sound side. He is unable to produce the slightest voluntary motion in the diseased limb, except by raising his pelvis. The affected member, when put into action by external force, is quite loose, and plays about as if it were hung in wires at the hip-joint. He has been ten years afflicted with this disorder, and imputes it to a weakly state of health, the consequence of a fever contracted in his native island of Jamaica, when he was only two years old.

On examining the spine with Dr. Hutchinson, we found a slight curve between the shoulders. All the lumbar vertebræ had sunk downwards, making the inward curve and bending a little towards the right. The transverse processes on the left side were too much risen, and turned into the left loin. There is an extensive hollowness in the right loin. All around the skin is unusually tender and flaccid.

He is often troubled with difficulty in voiding his urine, and has been supposed to suffer from the gravel. The bowels are constipated. The stools have a very offensive smell and blackish colour. In other respects he is very well.—*Feb.* 5*th*, 1821.

The depression of the spine and hollowness in the loins having been nearly removed at the last visit, he was enabled, before I left him, to move his toes a little; a pleasure which he had not enjoyed for the last ten years. When I called this afternoon, I found less of the speckled appearance of the skin. It was warmer and more sensible. He had made water several times with freedom and ease. The stools looked better, and their disagreeable smell had abated.—*Feb.* 7.

The skin in the loins and all over the limb has recovered its proper feeling. The warmth and natural colour are entirely restored. He moves his toes and ankles with a good deal of freedom. He has got a little motion in his hip-joint; that of the knee is scarcely perceptible. He often feels something stiff, like a tense cord beginning as it were below the foot, running behind the inner ankle, and extending upwards to the ham; from thence passing in the inside of the thigh to the groin.—*Feb.* 15.

He can use the hip-joint so as to raise and draw up the affected limb with great ease; he can also move the joints of the knee, ankle, and toes; but the exertion is fatiguing. He has often prickly itching, and other indescribable sensations, in different parts of his leg and thigh. He has at times great numbness near the neck of the bladder, and very distressing pains in the glans penis.—*Feb.* 19.

He has acquired the perfect use of all the joints in his right leg and thigh. He can draw them outwards with ease, but finds more difficulty in pulling them towards the other limb and in rolling them inwards. The health is in all respects good; and he has grown several inches since he first lay down.—*April* 10*th*.

The strength and motions of the limb have improved considerably since the last report; he can raise it nearly to a right angle with the body, and draw it forwards with ease. These various actions are freely performed, but, after a few repetitions, they induce uneasiness and fatigue. The urine and stools have been natural for some time past. He no longer feels any distressing sensations about the groin or bladder. He is in all respects well and comfortable.—*May 25th,* 1821.

*Ninth Case.*

Miss N. O. aged fourteen years last March, of a florid complexion and delicate habit, has for the last six years been afflicted with a lateral curve, which has been gradually increasing. The vertebræ can be distinctly felt passing under the right shoulder-blade, which stands a good deal above its fellow. She can strike the two scapulæ together with considerable force. Lower down, the vertebræ are irregular in height, in their direction, and in their distances from each other. All the lumbar and lowest dorsal bones are too protuberant, and are considerably arched. The ribs on the right side are depressed. On the left, the back is unusually hollow. Forwards the chest is peaked. In front, the ribs are too much risen; those of the right side are sunk downwards. Owing to this disposition of the breast, the left mamma is much fuller than the other. The right shoulder-top is visibly higher than the opposite one; and the foot on that side requires a thicker-soled shoe to make her limbs of equal length. Menses and bowels are regular; her health is good. The back measures twenty-one inches.—An elder sister, affected with the same kind of deformity, having lately died of pulmonary consumption, and, as her family believe, in consequence of it, their solicitude is increased to get her delicate form restored, to preserve her from a similar fate. *Nov.* 10, 1820.

The vertebræ are more regular in their height and direction. The lumbar bones are still raised, but the arch has wholly disappeared. The hollowness is become equal on both sides. The chest in front is quite open. The ribs are more forward on the left than towards the right. Mammæ are nearly alike; and there is little difference in the shoulder-tops. The back has sensibly increased, being now twenty-two inches long. Her health is in all respects very good.—*Dec.* 14.

The vertebræ are quite regular; the lumbar bones have sunk into their natural places. On the right side the ribs are still too high, and the opposite spine is a little crooked.—*Feb.* 25.

The ribs are nearly equal on both sides, and the spine is scarcely bent. She is in the best possible health.—*April* 10.

The natural form of the spine and chest has been sometime restored. She is in a very good state of health, and in excellent spirits. Her constitution being peculiarly delicate, she is advised to continue the horizontal position for some time longer. *May* 24, 1821.

### *Tenth Case.*

Miss P. Q. a tall, well-formed, delicate lady of fifteen, complains of being soon fatigued with walking or dancing, in both of which recreations she used to take great delight, and to persevere in them with much pleasure for several successive hours. She is subject to hysterics from exercise, to frequent attacks of numbness, coldness, and spasmodic twitchings in her arms and legs. These symptoms having come on within the last three months, I was consulted this forenoon, under an idea that they originated in the spine.

On examination, I found a large swelling of the right shoulder, and another in her left loin, with marked curves in the upper dorsal and lumbar vertebræ. There were corresponding depressions in the left side and right loin. Menses have lately been frequent and profuse.—*Jan.* 6, 1821.

The health is in all respects good, and the deformity is entirely removed. She indulges in music, and enters with moderation into the various recreations of this gay capital.—*Feb.* 21.

### *Eleventh Case.*

Master Burton, aged fifteen months, of a sanguine temperament, and scrofulous constitution, is of a small size for his age. He is backward in walking and talking. When the mother carries him, which is almost constantly, he reclines on her left arm and bosom, with his right side lying against her person. In this posture, his right arm being fixed, he necessarily employs his left hand on all occasions.

On examination, I found his back-bone between the shoulders bent towards the left, and in the loins towards the right. His right side is forced inwards, by the weight of his body placed over it. His left bosom stands very protuberant, showing a marked difference between the two sides of the chest.—*Jan.* 2, 1821.

I have been insensibly led, by a concurrence of accidental circumstances, to entertain some peculiar opinions on the subject of spinal distortions and their distressing consequences. These I propose to lay before the public, in a series of Essays, as my leisure and opportunities will permit, till I shall have fully explained my ideas of the causes and treatment of the disease. After having practised as a physician, and treated spinal complaints upwards of thirty years, according to the usual routine, I was consulted for a married lady, who roused my attention and interested my feelings in an extraordinary manner. She had already submitted to issues and the recumbent posture for more than twelve months, under the care of eminent metropolitan practitioners. As she had derived no benefit, I resolved upon the substitution of other expedients. A period of three months having been unavoidably suffered to elapse before any steady course could be adopted, I employed the time in a careful examination of the prevailing doctrines, and was brought to entertain doubts of their consistency and truth. Under this uncertainty, I suggested a trial of frictions and pressure to the curved vertebræ, and had the satisfaction ultimately to force them back again into their natural stations. In the following summer, I was of equal service to four other invalids. The succeeding spring afforded opportunities to prosecute my plan with several fresh cases. Hitherto, agreeably to professional etiquette, I had merely directed and superintended the new method, leaving its execution to the domestic attendants. I began at length to suspect that, from their total ignorance of anatomical knowledge, the progress was greatly impeded, and often unnecessarily interrupted. Impressed with this idea, I deemed it my duty, in order to ascertain the truth, to become an operator myself: the consequent success having fully justified the suspicions entertained, and satisfied my most sanguine anticipations in the more speedy and complete recovery of my patients, I have ever since continued to take an active part in the treatment. In compliance with the sage maxim of the learned Celsus, that physicians are to cure *cito, tuto, et jucunde,* and fortified also with the most ample licence and authority contained in my doctor's diploma, obtained at the University of Edinburgh, I shall not hesitate in future to interpose my own personal exertions, so long as I am encouraged to believe they conduce towards the more speedy recovery of my patients; leaving it to the learned professors and *senatus academicus* of my Alma Mater to defend their own regulations, should they be assailed from any quarter. Whatever may be the result, I am fully convinced that ere long I shall be entitled, through this very mode, to the gratitude of my brethren for having introduced them to a new branch of practice, in the enjoyment of which they will have the unspeakable satisfaction of removing personal deformity, restoring the use of crippled limbs, and preventing the ravages of many fatal complaints which have hitherto baffled the most skilful and experienced.

The presence of spinal maladies may be discovered, in most instances, by merely looking at the countenance, which appears anxious, distressed, aged, and care-worn. Several of these symptoms were observed in the face of Mary Rafter, and they grew fainter as the distortion abated. Professor Rust has detailed thirteen of these cases, where the atlas and second vertebra were carious. They are strikingly illustrated in an accompanying print. In it the features are made to exhibit the most lively agony and distress. Cephalœa is a common attendant. The pains may be internal or external, and are by no means confined to any particular spot; they invade sometimes one part of the head, sometimes another; the violence may continue many hours without intermission, or go off entirely in a few minutes. The invasion and abatement are equally sudden. These pains, like those of the tic douloureux, are distinguished from most others, by their leaving the patient cheerful and easy. During the intervals he expresses no apprehension or alarm about their return, and seldom mentions them.

We shall have no difficulty in understanding the cause of this species of cephalœa, or of the distortion of countenance, when we consider the intimate association which takes place in the neck between the great sympathetic, the par vagum, recurrent nerves, spinal accessary, glosso pharyngæus and laryngæus, the portio dura of the seventh, the last of the cerebral, first and second spinal, nerves, &c.

Some of these nerves are connected with branches distributed upon the different parts of the head, and form a medium of intercourse between them and the great sympathetic. These are chiefly the pes anserinus and other ramifications of the fifth pair, the portio dura of the seventh, and upper cervical nerves. Hence we can easily understand how a distorted state of the vertebral pillar produces cephalœa, and is displayed in the countenance.

Deafness is another attendant upon spinal maladies, as I shall have occasion to notice in the series of cases which it is my intention to lay before the faculty. The explanation of this symptom will readily occur to the anatomical reader, when he reflects upon the communication which subsists between the great sympathetic and auditory nerve, the portio mollis of the seventh pair, through the portio dura of the same trunk.

*7, Holles-street, Cavendish-square; May* 1821.

## Figures and Tables

**Figure f1:**